# A Tactile Sensor Decoupling Process

**DOI:** 10.3390/s18103515

**Published:** 2018-10-18

**Authors:** Yuyun Xu, Xuekun Zhuang, Guangyu Hu, Hongqing Pan, Feng Shuang

**Affiliations:** 1 Institute of Intelligent Machines, Chinese Academy of Sciences, Hefei 230031, China; xuyuyun@mail.ustc.edu.cn (Y.X.); zhuangxk@mail.ustc.edu.cn (X.Z.); hugy@mail.ustc.edu.cn (G.H.); 2 Department of Automation, University of Science and Technology of China, Hefei 230026, China; 3 College of Electrical Engineering, Guangxi University, Nanning 530004, China

**Keywords:** homotopy, tactile sensors, trust-region dogleg, decoupling process

## Abstract

An improved hybrid homotopy method is proposed to decouple the multi-input model of tactile sensors. The time-embedded homotopy algorithm is proved to be very suitable for solving the problem. Three tracking factors that control the efficiency of the algorithm are studied: tracking operator, stepsize, and accuracy. Trust region methods are applied to track the zero paths instead of the traditional differential algorithm, and a periodic sampling method is proposed to improve the efficiency of the algorithm. Numerical experiments show that both the robustness and accuracy have received a huge boost after the hybrid algorithm is applied.

## 1. Introduction

The flexible tactile sensor array is very important in the field of robot skin. In the latest research, the development of tactile sensors has mainly focused on capacitive sensing, piezoresistive sensing, piezoelectric sensing, etc. [1,2,3]. A 3D tactile sensor based on the different capacitive principles has been reported in Chu et al. [4]. Strohmayr et al. [5] fabricated a flexible tactile sensor for robotic hands based on a crossed-wire approach. Bal et al. [6] have developed a high-sensitivity artificial skin based on transparent elastic films of carbon nanotubes. Koc et al. [7] developed a piezoelectric tactile sensor that can detect both force and force distribution by using PVDF material. Oballe-Peinado et al. [8] presented a realization of the electronics of a tactile sensor suite mounted on a dexterous robotic hand by using FPGAs for scanning and pre-processing tactile data.

At present, a coupling relationship general exists in the three-dimensional tactile sensor and it is always nonlinear. Therefore, the study of decoupling algorithms makes great sense in the field of multi-dimensional sensors. A lot of methods have been tried to decouple the multi-dimensional information of sensors. Baglio et al. [9] used the fuzzy logic method to identify the characteristic signals in the unknown surface of a tactile sensor system. Petra et al. [10] applied a BP neural network to improve a distributed tactile sensor system, and realized the decoupling circuit of the BP network based on hardware structure. Schram et al. [11] proposed the optimal parity vector method to calibrate and diagnose array sensors. Ma et al. [12] presented a robust static decoupling algorithm for multi-dimensional force sensors based on support vector regression. Wang et al. [13] studied the decoupling method based on a RBF neural network for tactile sensors.

In order to solve the problem of nonlinear coupling in the tactile sensor model, a homotopy method [14,15,16,17] was introduced. A time-varying variable was embedded into the decoupling process to avoid the problem of multiple solutions, which has universal significance for this type of model. Then the high dimensional decoupling problem can be transformed into a zero path tracking problem by the improved homotopy algorithm, which is a novel and effective method to solve the decoupling problem in the tactile sensor model.

## 2. The Model of Tactile Sensors

### 2.1. Tactile Sensor

**Figure 1:**
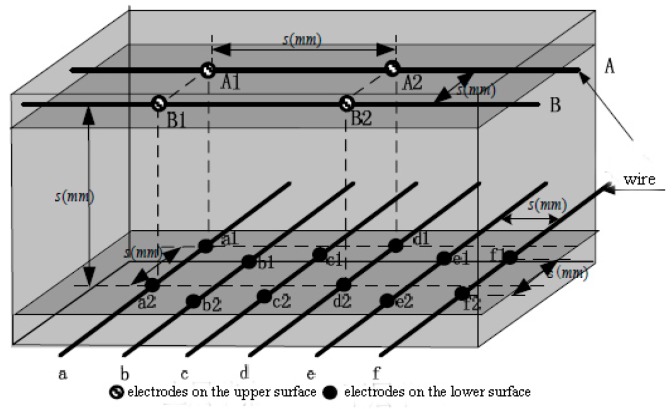
The structure of the sensor nodes array.

The sensor adopts a two layer wiring structure to construct the signal collection network, which is shown in Figure 1 [18]. The resistance between the electrode nodes in the upper and lower wires can be detected when a force is exerted on the surface of the tactile sensor. Then by analyzing the resistance matrix by the decoupling algorithm of the sensor, we can get the size of the force on the sensor. In order to reduce the analytical difficulty, we assume an injection moldable flexible tactile sensor developed by a flexible piezoresistive conductive rubber under excellent conditions. According to the isotropic, linear and non-viscoelastic elastomer ideal piezoresistive properties of the conductive rubber, the resistance between any two electrode nodes in conductive rubber is proportional to the distance between two points. Under a small deformation, the conductive rubber obeys Hooke’s law: F=k∗Δs, where F is the external force on the conductive rubber, and Δs is the deformation of the conductive rubber.

### 2.2. The Numerical Model of Tactile Sensor

**Figure 2:**
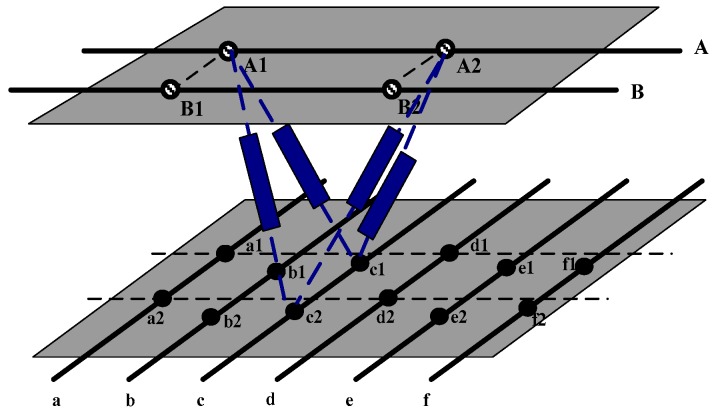
The parallel resistance model when no deformation has occurred.

**Figure 3:**
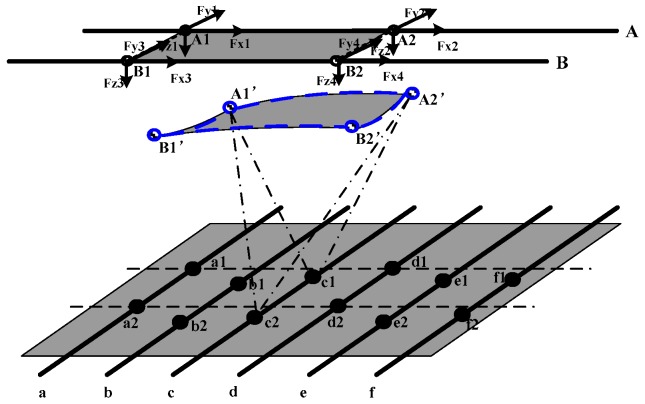
The parallel resistance model with an applied deformation.

In order to describe the model in more details, two schematics are presented. Figure 2 shows the upper surface of the sensor when no deformation has occurred. When an external force is applied to the upper surface of the sensor, the position changes of the electrodes are shown in Figure 3, and the decoupling task is to track the deformation of these points and try to infer the deformation of the upper surface. The parallel resistance between wire A and c shown in Figure 2 can be written as:

(1) $$R_{Ac}=R_{A_{1}c_{1}}\|R_{A_{1}c_{2}}\|R_{A_{2}c_{1}}\|R_{A_{2}c_{2}}.$$

when *N* electrodes are embedded on each wire of the upper surface, and M electrodes are embedded on each wire of the lower surface. In order to decouple the position coordinates after loading of the electrodes on the upper surface (that is, 3 × *N* variables are needed to solve the problem), a system equation that contains 3 × *N* variables should be constructed to acquire the coordinates of the *N* electrodes on an upper wire. In order to avoid the phenomenon of the appearance of overdetermination or underdetermination, the 3 × *N* resistances equations between a wire of the upper surface and 3 × *N* wires of the lower surface are needed in the modeling process. The system equations of the parallel resistance model are as follows:

(2) $$\sum_{j=1}^{M}\;\sum_{i=1}^{N}\frac{1}{\sqrt{{\left(x_{i}-\bar{x_{jk}}\right)}^{2}+{\left(y_{i}-\bar{y_{jk}}\right)}^{2}+{\left(z_{i}-\bar{z_{jk}}\right)}^{2}}}=g\frac{1}{R_{k}},k=1\ldots 3N.$$

where, *i* and *j* represent the label of an electrode embedded into a wire on the upper layer and the lower layer, respectively, *k* represents the quantity of the wires on the lower layer, $x_{i},y_{i},z_{i}$ represent the three subcoordinate components of the *i*-th electrode to be decoupled, $\bar{x_{jk}},\bar{y_{jk}},\bar{z_{jk}}$ represent all the coordinates of the electrodes embedded into the lower layer and represent the coordinate components of the *j*-th electrode embedded into the *k*-th lower layer. In this article, we treat the decoupling process of the high dimensional model as an engineering optimization problem, and the goal is to find a method that can decouple this model rapidly and with acceptable accuracy. An improved homotopy method is introduced to solve this problem in the next section.

## 3. Improved Homotopy Method

In this section, an improved homotopy method is proposed. When facing this multi-input nonlinear model, the first thing to be considered is how to maximize the use of information that would be gained from the external circuit during the decoupling process, which can be met in the homotopy method. When trying to decouple this problem directly, there will appear a series of very intractable problems including singularity, robustness, multiple solutions, etc. caused by the non-linear complexity of the problem. A time-varying variable is embedded into the homotopy method and the efficient zero path tracking operator is selected in order to fix the high dimensional nonlinear system.

### 3.1. Homotopy Method

**Figure 4:**
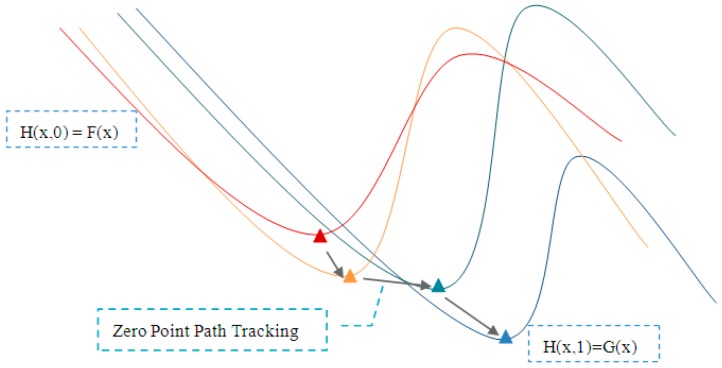
A series of homotopic deformations.

Homotopy is a concept originally used in topology, which says that two continuous functions from one topological space to another are called “homotopic” and if one can be “continuously deformed” into the other, such a deformation being called a homotopy between the two functions (Figure 4).

The Newton method and its variations are widely used for solving a system of nonlinear equations such as:

(3) $$F(x)=0,\mathrm{where}\;F:R^{n}\to R^{n}.$$

Due to their computational efficiency, these methods can achieve super linear convergence if the initial starting point is within a certain neighborhood of the solution. Unfortunately, this type of method often fails if the initial guess is not sufficiently close to the solution, or if singular points are encountered. In other words, these methods are locally convergent and cannot provide guarantees of obtaining solutions [19,20,21].

Homotopy methods have been developed to overcome the disadvantages of Newton-type methods. Homotopy methods are usually slower than the Newton-type methods, but they are effective for solving difficult systems of nonlinear equations for which a good starting point is hard to find and the Jacobian matrix is in bad condition.

Stated briefly, homotopy-continuation methods consist of the following parts: to solve Equation (3), one should define a homotopy function $H:R^{n}\times R^{n}\to R^{n}$ such that *H*(*x*,1) = *F*(*x*) and *H*(*x*,0) = *G*(*x*) where $G:R^{n}\to R^{n}$ is a smooth mapping with known solutions. Typically, one may choose a convex homotopy function such as:

(4) H(x,λ)=λF(x)+(1−λ)G(x)

and attempt to trace an implicitly defined curve in $H^{-1}\left(0\right)$ from an initial guess $\left(x_{0},0\right)$ to a solution point $\left(x^{*},0\right)$. If this succeeds, then a zero point of $x^{*}$ is obtained.

### 3.2. Imbeded Time-Varying Variable

Aimed at the problem of a numerical model of the tactile sensor, this article introduces a time-varying variable *t* into the homotopy function instead of using an abstract mathematical variable λ Here the problem will not be decoupled directly, and the resistance signals corresponding to the deformation process of the tactile sensor are collected as a guide to solve the homotopy function. The original system of equations is written as:

(5) $$\{\begin{array}{c} f_{1}\left(x_{1},x_{2}\ldots x_{N}\right)=g\frac{1}{r_{1}}\\ \ldots \\ f_{k}\left(x_{1},x_{2}\ldots x_{N}\right)=g\frac{1}{r_{k}}\\ \ldots \\ f_{N}\left(x_{1},x_{2}\ldots x_{N}\right)=g\frac{1}{r_{N}} \end{array}.$$

Assuming that the time variable is *t*, Equation (5) can be simplified as:

(6) F(X(t))=G(R(t)).

where $X(t)=[x_{1}(t),x_{2}(t)\ldots x_{N}(t)]$ and $R(t)=[r_{1}(t),r_{2}(t)\ldots r_{N}(t)]$, *g* is a rubber material property-related variable which can be regarded as a constant under ideal conditions. A homotopy function can be constructed as:

(7) H(X(t),R(t))=F(X(t))−g(t)G(R(t)).

**Figure 5:**
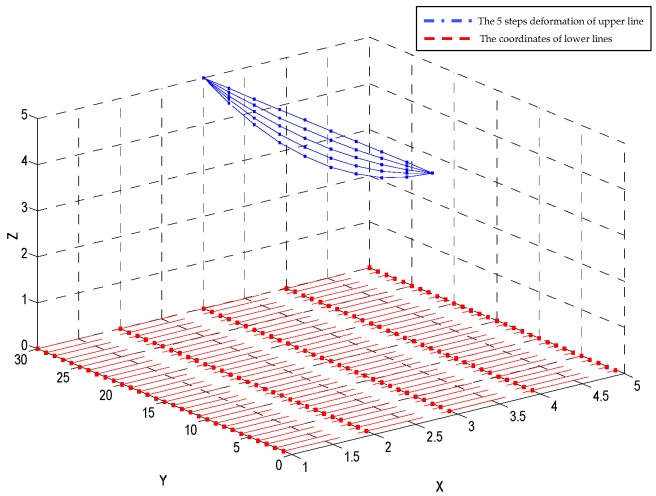
Using five steps to split the deformation of a tactile sensor.

**Figure 6:**
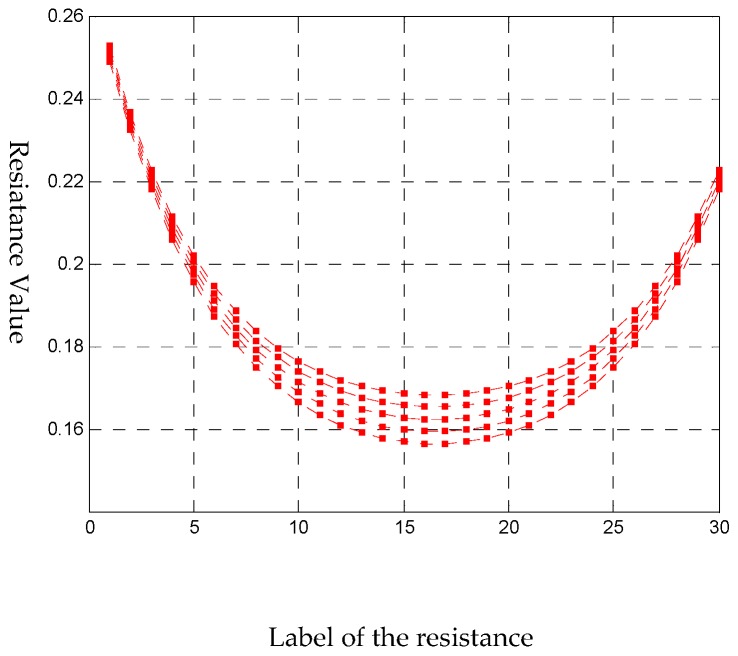
The corresponding 30 resistance signals of each step (five steps).

The deformation process of the tactile sensor is shown in Figure 5. The corresponding resistance signals should be obtained from the external circuit. When a deformation happens, a series of resistances shown in Figure 6 are stored to guide the algorithm to decouple the deformation.

If we split the deformation process into five steps, the corresponding resistances are [R(0),R(Δt),R(2Δt)…R(4Δt)]. In the improved homotopy method, the coordinates without any deformation are used as the initial solution of the homotopy function (Equation (7)), namely H(X(0),R(0))=F(X(0))G(R(0)) which can be very easily satisfied. When the first resistance R(Δt) is applied, the system function is H(X(0),R(Δt))=0 which can be seemed as a homotopy of H(X(0),R(0))=0 with a stepsize Δt. If the initial solution is X(0), then the solution of H(X(0),R(Δt))=0 can be reached easily because the distance between the zero points are very short. In this process, we applied a method like Lahaye’s iterative continuation approach. In the following part we should choose an effective zero path tracking operator to complete the intermediate iterative process, we will set 10 electrodes on each line at the upper layer of the tactile sense and 10 lines on the upper layer, that means 30 variables in the system equations to form an 10 × 10 electrodes system to restore the original surface of the tactile sensor.

### 3.3. Diagram of the Improved Homotopy Method

Based on the descriptions above, here a flow diagram of the improved homotopy algorithm is given in Table 1.

## 4. Zero Path Tracking Operators

Any ordinary iterative method is capable of accomplishing the homotopy deformation zero tracking task. In order to pick an efficient and robust method, we tested three kinds of efficient operators, including GN (Gauss-Newton) method [21], LM (Levenberg-Marquard) method and trust-region dogleg (TR-dogleg) method [22]. These three methods are all famous for their high efficiency and robustness. The number of calls of the objective function is chosen as the efficiency measure of the algorithms.

**Figure 7:**
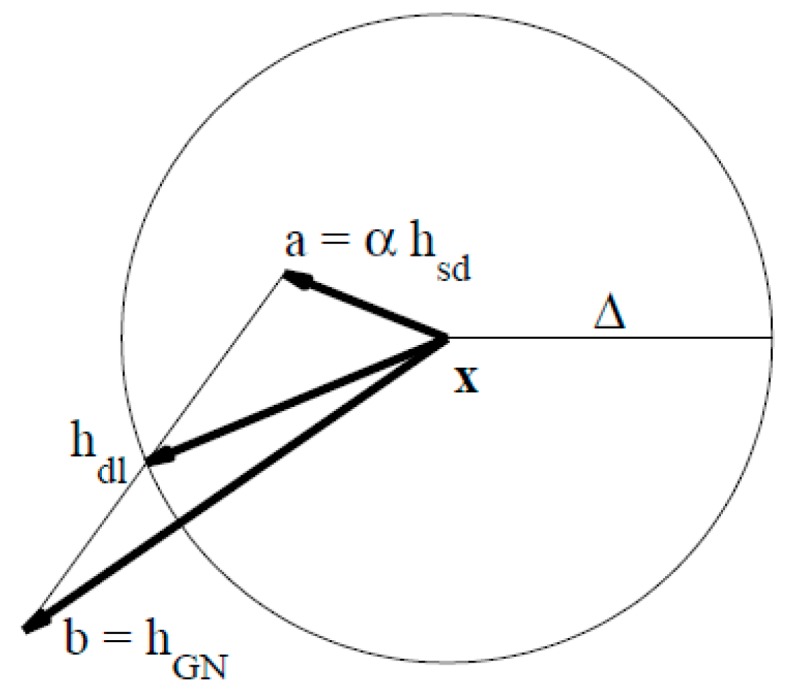
Construction of the TR-Dogleg step.

Figure 7 gives the construction of the TR-dogleg step. In the TR-dogleg operator, step d is constructed from a convex combination of a Cauchy step (a step along the steepest descent direction) and a Gauss-Newton step for *F*(*x*). The Cauchy step is calculated as:

(8) $$d_{sd}=-\rho J{\left(x_{k}\right)}^{T}F(x_{k}).$$

where, ρ is an optimized value to decide the best step and $J\left(X_{k}\right)$ represents the Jacobian matrix of the equation. The Gauss-Newton step is calculated by solving:

(9) $$J\left(x_{k}\right)d_{GN}=-F(x_{k}).$$

In order to calculate the trust region radius, $\mathrm{if}\;\|h_{\mathrm{gn}}\|\leq \Delta$, then $h_{\mathrm{dl}}:=h_{\mathrm{gn}}$, $\mathrm{else}\;\mathrm{if}\;\|{\alpha h}_{\mathrm{sd}}\|\geq \Delta$, $h_{\mathrm{dl}}:=\left(\Delta /\|h_{\mathrm{sd}}\|\right)h_{\mathrm{sd}}$. If the two conditions are not met, then we use Powell’s constructor:

(10) $$h_{\mathrm{dl}}:={\alpha h}_{\mathrm{sd}}+\beta (h_{\mathrm{gn}}-{\alpha h}_{\mathrm{sd}}).$$

Table 2 shows the efficiency of the three tracking operators (as an average of 10 numerical experiments). The iteration termination conditions of the first *N* − 1 steps are set as Tol_Fun (the tolerance of function) = 1 × 10^−6^, Tol_X (the tolerance of x) = 1 × 10^−6^, and MaxIter (maximum number of iterations) = 100, and those of the last step are set as Tol_Fun = 1 × 10^−12^, Tol_X = 1 × 10^−12^, and MaxIter = 1000. Here, we can see that the dogleg method is more efficient than LM method, but less efficient than Gauss-Newton method.

Table 3 gives a comparison of the accuracy of the three methods. We can see that LM method has the best accuracy and GN has a relatively poor performance. When the number of sampling steps is small (such as 5, 10 steps), the GN method cannot even overcome the singularity problem. The performance of the dogleg algorithm is between LM and GN methods, with acceptable accuracy. In order to meet the requirements of real-time during the decoupling process of the tactile sensor, it is worth to sacrifice some accuracy in an homotopy intermediate step, so long as the null point path can be tracked as closer and quicker as possible at the last step. Taking all these factors, we decided to make a compromise to choose the trust region dogleg operator to accomplish the zero path tracking.

These numerical experiments show that the dogleg operator can perform quite efficiently and with acceptable accuracy, which is very important during the zero path tracking. However, high precision at intermediate steps is unnecessary and time-consuming. In next section, we will show how to relax the accuracy to improve the efficiency of the algorithm.

## 5. Tracking Stepsize and Accuracy

The effect of tracking stepsize and accuracy on the homotopy methods for tactile sensors is studied in this section with the tracking operator being the dogleg which is shown to be appropriate for homotopy decoupling algorithms for tactile sensors. The whole tracking process could be split into *N* steps with uniform stepsize. The stepsize is directly related to the efficiency of the decoupling algorithm. Excessive sampling steps will increase robustness of the algorithm, but it also leads to a lot of unnecessary calculations, and reduces the efficiency of the algorithm. On the other hand, if the sampling is too sparse, which means guiding information is not enough in the middle of the tracking interval, it would cause the singularity problem. Another trick to improve the efficiency of the homotopy algorithm for tactile sensors is by decreasing the tracking accuracy of the first *N* − 1 steps, while only keep the *N*-th tracking step accurately as required. It is necessary to study the interplay of stepsize and accuracy.

### 5.1. Stepsize of the Tracking

With the increase of the sampling steps, more information will be collected from the intermediate process to enhance the accuracy and robustness of the algorithm. As shown in Table 4, the calls of the objective function of the first *N* − 1 steps will decrease with the increase of the sampling steps, and when *N* > 100, the number of function calls become stabilized gradually, but the accuracy doesn’t improve too much. However, another problem appearing is that the time-consumption increases rapidly with the increase of the sampling steps. These factors combine to give a relative trade off choice, both for the accuracy and the computing complexity. The test results support about 10 steps in the parallel resistance model is good choice when 10% deformation is applied.

**Figure 8:**
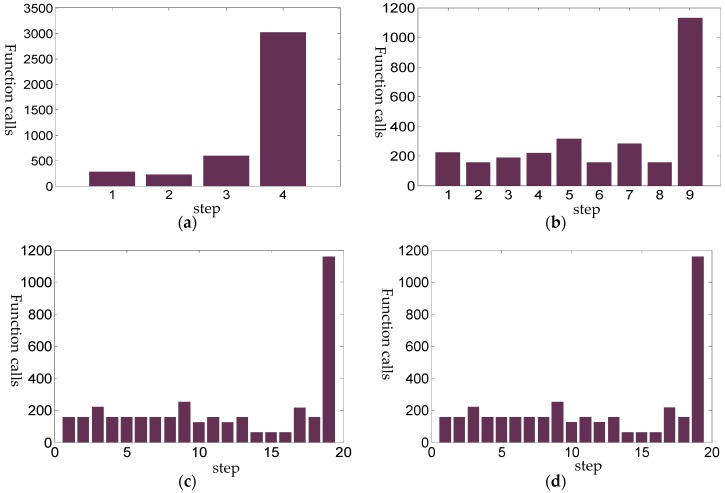
The distribution of the function call numbers for the first *N* − 1 sampling steps. (**a**) Sampling steps of 5; (**b**) Sampling steps of 10; (**c**) Sampling steps of 20; (**d**) Sampling steps of 50.

In order to observe the intermediate tracking steps more clearly, Figure 8 gives the distribution of the objective function calls under sampling steps of 5, 10, 20 and 50, respectively. Figure 8 shows that if less sampling steps are used, more objective function calls will be needed to maintain the accuracy.

### 5.2. Accuracy of the Tracking

In order to improve the efficiency of the algorithm further, some certain trade-offs should be considered to reduce the sampling steps of the intermediate zero path tracking process. Here we consider another strategy which relaxes the accuracy of the tracking operator except for the last step. The resistance sampling method introduced above is based on uniform sampling, in which a lot of redundant information may exist restricting the efficiency of the algorithm. Here, the main parameter to control the homotopy tracking step is the first-order optimality measure, i.e., a measure of the distance between the tracked point and the ideal point. The solution will be terminated when the first-order optimization is less than the iterative termination condition Tol_Fun. In smooth unconstrained problems $\min_{x}f\left(X\right)$, and the first-order optimization measure may be defined as $\mathrm{First}-\mathrm{order}\;\mathrm{optimality}\;\mathrm{measure}\;=\max_{i}\left|{\left(\nabla f\left(x\right)\right)}_{i}\right|={\left\|\nabla f\left(x\right)\right\|}_{\infty }$. This measure of first order optimality is based on the familiar condition for a smooth function to achieve a minimum: its gradient must be zero. For unconstrained problems, when the first-order optimality measure is nearly zero, the gradient of the objective function is nearly zero, so the objective function could be assumed nearly minimized.

**Figure 9:**
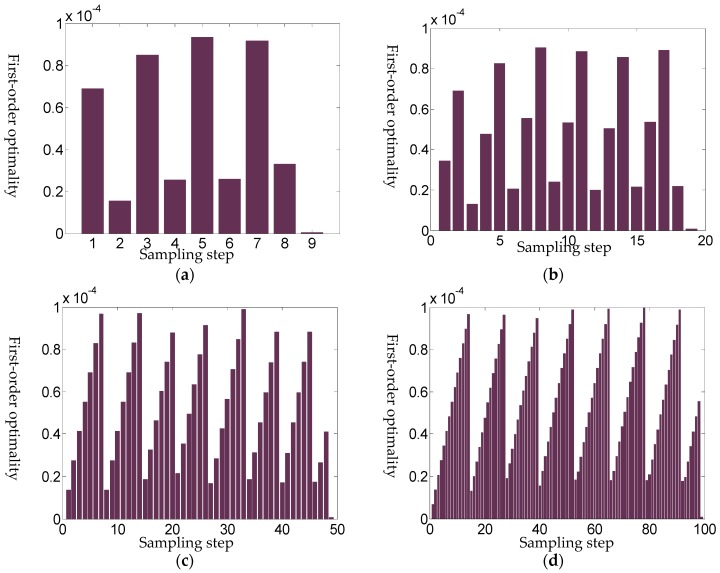
First-order optimality with different sampling steps under loose intermediate iteration termination condition10^−4^. (**a**) Sampling steps of 10; (**b**) Sampling steps of 20; (**c**) Sampling steps of 50; (**d**) Sampling steps of 100.

The first test is to relax the iteration termination condition of intermediate tracking steps from 10^−6^ to 10^−4^. Figure 9 shows the first-order optimality with different sampling steps under loose intermediate iteration termination condition 10^−4^. When offering a new guiding resistance to solve the homotopy equations, the new zero point is still fairly close to previous one, which leads to the algorithm reaching the iteration termination condition and skipping the tracking directly. With the increase of the number of hops, when the error accumulation breaks the termination condition, the algorithm will solve the trust region operator again. This periodicity phenomenon becomes more obvious when a higher sampling frequency is applied. As can be seen from Figure 9c, when the sampling frequency (step) reaches 50, the first-order optimization shows a significant periodicity which implies that information redundancy really exists in the zero path tracking process.

It is not necessary to track the redundant information. Through the above numerical simulations, it is known that the cyclical phenomenon depends on specific parameter of decoupling equations. For the parallel resistor model with 10% smooth deformation, when 50 steps are applied and the iteration termination condition is 10^−4^, the cycle T of first order optimality is about 7. Figure 9b–d indicate that the cycle is nearly proportional to the number of sampling steps. It means that in a cycle T, the intermediate sampling step is redundant and can be neglected, while the zero path tracking accuracy would not be greatly affected. From the above analysis, we can get a method to improve the efficiency of the homotopy algorithm. At the beginning, we use the peripheral sampling circuit to get a lot of resistance pilot signal. This is of relatively low cost, because the sampling frequency of the circuit can be relatively high (KHz level). Then, we let the homotopy method perform a uniform sampling step tracking (assuming N resistance signals), and relax the iteration termination condition gradually to acquire a rough homotopy cycle T. After that, in the case of a certain magnitude of the pressure, the uniform sampling is abandoned, and a series resistance value of the end of the cycle is selected, such that the homotopy tracking steps change from *N* steps into *N*/T steps. This strategy improves the efficiency of the algorithm greatly.

**Figure 10:**
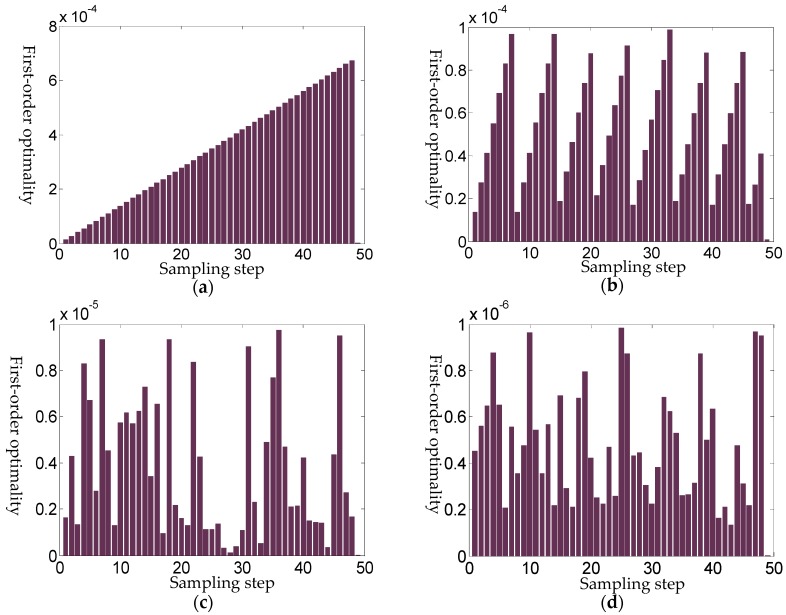
Different cycle of first-order optimality shows with respect to different intermediate iteration termination conditions, with 50 sampling steps. (**a**) Termination condition of 10^−3^; (**b**) Termination condition of 10^−4^; (**c**) Termination condition of 10^−5^; (**d**) Termination condition of 10^−6^.

It should be noted that the crucial procedure is how to relax the iteration termination condition. Figure 10 shows different cycle of first-order optimality shows with respect to different intermediate iteration termination conditions. If the termination condition is more relaxed, the error accumulation phenomenon is more obvious, and the periodicity is stronger. If the termination condition is relatively harsh, cyclical phenomenon tends to disappear. When the termination condition is too loose, the entire track would stay in only one cycle, while if the termination condition is too harsh, then periodically would almost disappear, and the algorithm almost returns to the uniform traversal tracking steps.

**Figure 11:**
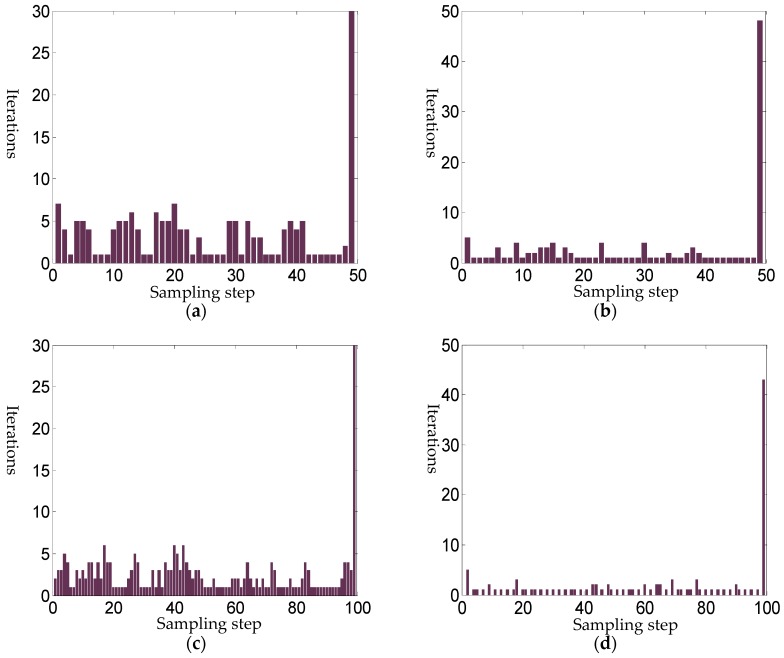
Iterations of the improved algorithm under different sampling conditions. (**a**) 50 steps uniform sampling tracking; (**b**) 50 steps periodic sampling tracking; (**c**) 100 steps uniform sampling tracking; (**d**) 100 steps periodic sampling tracking.

A comparison is given to show the efficiency of the uniform sampling method in Figure 11a,c, and the periodic sampling algorithm in Figure 11b,d. For the periodic sampling algorithm, the intermediate step iteration termination condition is relaxed to 10^−4^, while for the uniform sampling method, it is 10^−6^. The results show that the efficiency of the periodic sampling algorithm is greatly improved, which avoids almost half the number of iterations of the uniform sampling method. When 100 steps are used, the periodic sampling method uses 107 iterations in total, while the uniform sampling uses 248 iterations. Even when only 20 steps are applied in the uniform sampling, 139 iterations are still needed, and, the convergence speed is significantly slower than the skipping periodic sampling algorithm, as the intermediate termination conditions are harsh.

### 5.3. Accuracy of the Solution

In the homotopy method, its accuracy depends on the final step of the tracking, while the other step of tracking is just a means to reach the final solution. In order to measure the quality of the final solution, the first-order optimality is used as a measure of how close a null point x is to the optimal solution. For the decoupling of the tactile sensor model, the problem of solving nonlinear equations is transformed into a minimization problem in the dogleg operator, so the first order optimality should be as small as possible to ensure the system equations can get correct solutions with high accuracy.

**Figure 12:**
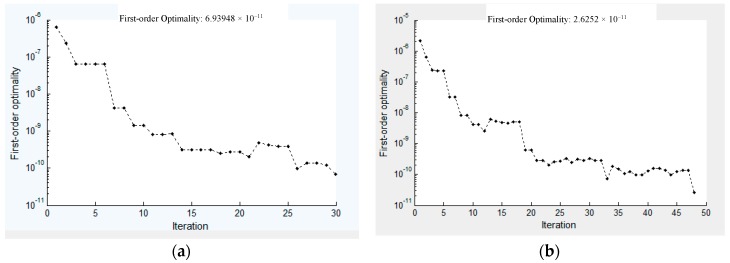
First-order optimality of the last step of the zero path tracking. (**a**) 50 steps uniformly; (**b**) 50 steps periodic.

When 50 steps uniform sampling are taken, the convergence of the last tracking step is shown in Figure 12, which shows that the convergence of the algorithm is very fast benefitting from the first *N* − 1 steps of the zero path tracking, and the precision can reach nearly 6.93 × 10^−11^, while the periodic sampling homotopy method precision is reaching 2.62 × 10^−11^, due to its lesser number of iterations, and the efficiency of the algorithm has been greatly improved.

**Figure 13:**
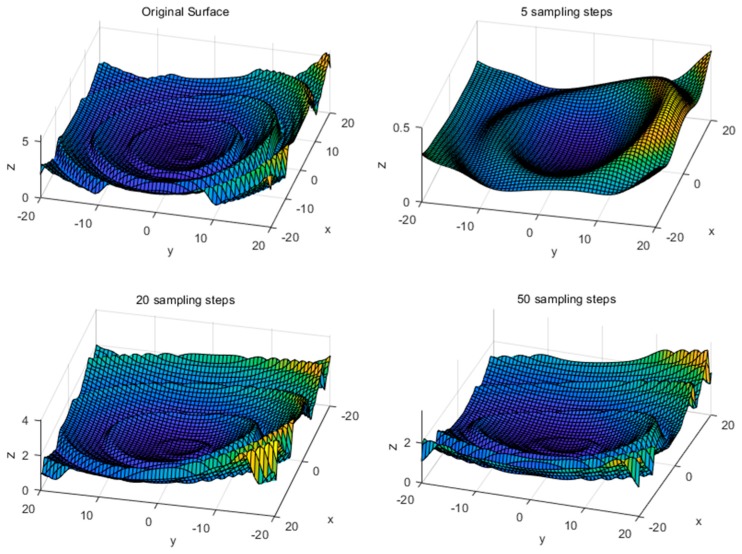
The decoupled surface of the tactile sensor under different sampling steps.

To see the actual decoupling effect, a deformation of 10% amplitude is applied on the top surface of the tactile sensor to simulate a pressing process. From Figure 13, if five steps are applied to restore the original deformation, a lot of information would be lost leading to a rough decoupling. However, when 20 more steps are applied, the decoupling results have shown high accuracy without obvious differences. The experimental results show that the sampling steps should be controlled within a reasonable range to improve the efficiency of the algorithm, and excessive sampling steps lead to information redundancy, which is unnecessary.

## 6. Conclusions

With the increase of sensor deformation, the mean square error of the decoupling algorithm increases and the result becomes unacceptable. When the array sensor is larger than six nodes in one line, the convergence speed of the genetic algorithm and evolutionary algorithm is slow and the error is large because of the large search space. When the sensor scale is larger, the ant colony algorithm can’t solve the problem. If the initial point is not properly chosen, the Newton-type iterative methods may converge to a local minimum of the optimization function rather than the solution of the nonlinear system. The complexity of the sensor mathematical model and the increase of its scale will not lead to the exponential increase in computational complexity of the homotopy algorithm. In this article, an improved homotopy method is proposed to solve the decoupling problem of the tactile sensor with high dimensions. Here, we do not try to directly solve the nonlinear problem, and the homotopy idea is combined to construct an algorithm to take good advantage of the intermediate resistance signals during the deformation process of the sensor. Simulations show that the intermediate resistance signals give a good guidance to simplify complexity of the problem. The sampling step of the improved homotopy algorithm is also discussed to improve the computational efficiency of the algorithm, and the dogleg operator is chosen to accomplish the zero paths tracking task based on Lahaye’s approach. Simulations show that when the intermediate iteration termination condition is relaxed, there will appear a periodic phenomenon of the first order optimality, and if periodic sampling is applied instead of uniform sampling, the efficiency of the algorithm can be improved without any loss of accuracy, although it should be mentioned that the time-varying homotopy algorithm is studied in relatively few quantitative problems at present, and a lot of mathematical properties of homotopy method require further study.

## Figures and Tables

**Table 1:** Steps of the improved homotopy method.

**Step 1.** Initialize the sampling frequency of the external circuit, namely determining the steps of the Homotopy tracking path. If there needs *N* steps in the tracking path, then the sampled resistances should be [R(0),R(Δt)…R(kΔt)…R((N−1)Δt)]. The original coordinates of the upper surface electrode is recorded as *X*(0).
**Step 2.** Put R((k−1)Δt) into the Equation (7), and run the *k*th zero point tracking operator dog-leg step to get the *k*th zero point X(kΔt). Use the current zero point X(kΔt) as the start point of next Homotopy step, and meanwhile put the R(kΔt) into the Equation (7) to solve the next zero point.
**Step 3.** Repeat Step 2 until the zero point X((N−2)Δt) is obtained, and now use iteration termination conditions of higher precision for the *N*th step, which can reach the final solution of the system equations.

**Table 2:** The efficiency of three tracking operators in homotopy algorithms.

Operator	LM (Times)	GN (Times)	Dogleg (Times)
5 steps	403.00	195.00	251.50
10 steps	403.00	102.30	220.29
20 steps	397.53	91.50	149.06
50 steps	388.48	80.50	104.90
100 steps	280.50	62.00	82.70

**Table 3:** Accuracy of three tracking operators in homotopy algorithms.

Operator	LM (%)	GN (%)	Dogleg (%)
5 steps	0.78	(Failed)	6.88
10 steps	0.037	(Failed)	0.19
20 steps	0.025	8.64	0.18
50 steps	0.0042	7.56	0.16
100 steps	0.0026	5.89	0.14

**Table 4:** Performance of the improved homotopy algorithm under different sampling steps.

Steps	Function Calls	Tol_X	Runtime (s)	Accuracy (%)
5	251.50	5.57 × 10^−12^	1.21	6.88
10	220.29	4.70 × 10^−12^	2.03	0.19
20	149.06	1.24 × 10^−12^	4.32	0.18
50	104.90	1.18 × 10^−12^	9.18	0.16
100	82.70	1.49 × 10^−12^	20.58	0.14
200	67.40	1.01 × 10^−12^	48.80	0.14
500	64.50	0.94 × 10^−12^	72.73	0.14
1000	63.40	0.91 × 10^−12^	271.58	0.14
